# Anterior subject positioning affects the maximal exerted isometric plantar flexion moment

**DOI:** 10.1371/journal.pone.0219840

**Published:** 2019-07-22

**Authors:** Savvas Stafilidis, Christoph Sickinger

**Affiliations:** Department of Biomechanics, Kinesiology and Computer Science in Sport, Institute of Sport Science, Sport, University of Vienna, Vienna, Austria; University degli Studi di Milano, ITALY

## Abstract

We examined the effect of increased anterior subject positioning toward the dynamometer’s footplate during maximal voluntary isometric contractions (MVCs) on the joint moment, rotation and rate of torque development (RTD). Fourteen subjects, with their hip flexed (110°) and knee fully extended (180°), underwent ramp maximal and rapid voluntary isometric plantar flexion contractions at 4 different positions (0, 3, 6 and 8 cm; randomized). At position “0 cm”, the foot was in full contact with the footplate; at the additional positions, the chair was moved forward. Body kinematics (VICON) and kinetics (HUMAC Norm, PEDAR) were captured synchronously during MVCs and RTDs. The results showed that the maximal exerted joint moment was significantly (p<0.01) increased by >32% from the 0-cm to 8-cm position (126 and 172 Nm, respectively); however, at the “6 cm” and “8 cm” positions, no significant difference was found. The joint rotation was significantly (p<0.01) reduced by >50% (from 15.5 to 7.1°; 0–8 cm). The maxRTD was only significantly higher at “6 cm” compared with the “0 cm” position. The time to reach maxRTD showed shorter tendencies for the “8 cm” position than for all other positions. The results indicate an underestimation of the plantar flexor maximal force potential with the current measuring technique. This could be critical in pre-post study designs where a 2-cm alteration in the chair position can induce an error of ~9% in the joint moment. The joint rotation could be reduced but not completely eliminated. For position standardization purposes, a pressure >220 kPa under the subject’s foot is needed to achieve the maximal joint moment. We discussed the possible origins (fascicle length, neural drive) of the increased joint moment.

## Introduction

The isokinetic dynamometer is a widely used [1–6] apparatus to examine the mechanical and morphological properties of the muscle tendon unit. In the literature [2,6,7], two major types of devices can be roughly categorized as “custom made” and “commercial dynamometer”. Most of the scientific research is being conducted in the second type, which is primary developed for rehabilitation and physiotherapy purposes [8]. Therefore, those devices have cushioning pads and more moving parts, and, consequently, are less rigid than the custom-made devices [4,8,9].

The nonrigidity of the commercial dynamometers induces drawbacks in the experimental results as reported in numerous studies. For example, Herzog (1988) [10] showed that the derived measured moment is different from the resultant joint moment in knee extension contractions; therefore, the author suggested caution when concluding about the muscle properties. The author further stated that factors that could influence the measuring results include gravitational forces, inertial forces, the elasticity of the dynamometer arm/foot system, the joint rotation and the axis misalignment. In another study, Arampatzis et al. (2004) [3] showed that, at the knee extension effort, misalignment of the knee joint axis could falsify as much as 17% of the exerted moment. The same group also reported in another study [4] that, at maximal plantar flexion contractions, the difference between the measured and resulted exerted moment could reach, on average, 6–10% (range 0.2–23%). The authors suggested that the difference was caused by the nonrigidity of the isokinetic dynamometer and the existence of the human body soft parts. Further studies [2,5] pointed out that, during plantar flexion contraction, an inevitable ankle joint angular rotation occurs that can influence the final results and, therefore, must be considered.

The nonrigidity of the dynamometer can also further influence the estimation of various mechanical and morphological parameters of the muscle tendon unit. For example, joint rotation [5] could shorten the fascicles and, therefore, would not operate on their optimum or dedicated length. Furthermore, the maximal activation assessment at maximal voluntary contractions, as measured using the twitch interpolation technique [1,11], could also be affected by the elasticity of the subject-dynamometer system, resulting in a systematically smaller twitch force [12]. Additionally, the nonrigidity of the dynamometer-subject system can have further implications on the estimation of the tendon’s mechanical (stress, strain or young modulus, stiffness) characteristics. In the case of longitudinal repeated measurements, an estimation error of the exerted moment could falsify the tendon’s mechanical characteristics and, hence, the intervention effectiveness.

Although attempts have been made to correct possible factors (joint rotation [3,5,13]; axis misalignment [3,5]; gravitational and inertial forces [3,5]; Co-Activation [2,3,14]) influencing the joint moment output during plantar flexion contractions, to the best of our knowledge, the effect of reduced device elasticity induced by a higher pressure on the foot to the joint moment output has not been examined yet. Therefore, the present study tackles the specific shortcoming of the nonrigidity of the dynamometer arm/shank-foot system, during maximal plantar flexion efforts. The main aim of this study was to identify if a higher pressure on the foot on the isokinetic apparatus can alter the muscle’s mechanical output (max. joint moment and rate of torque development) during plantar flexion contraction. Possible alterations in the ankle joint geometry during plantar flexion contractions induced from the subject’s positioning were also considered.

We hypothesized that a higher foot pressure will increase the maximal exerted moment and decrease the rotation of the ankle joint. Finally, we hypothesized that a higher foot pressure will also have an effect on the subject’s rate of torque development.

## Method

Eighteen healthy adult males initially participated in the study. Four participants could not complete the whole test battery and, therefore, were excluded from further analysis. Their mean (±SD) anthropometric parameters were as follows: age: 29.4 ± 6.1 years; body mass: 77.0 ± 9.8 kg; height: 181.4 ± 4.7 cm. They were randomly acquired at the Centre for Sport Science and University Sports in Vienna where they regularly participate in physical activity. All participants provided their written informed consent prior to participating in the study. The study was approved by the Ethical Committee of the University of Vienna (decision number 00264). All participants provided their written informed consent before initiation of the study. None of the participants had any major or recent musculoskeletal injury in the examined leg at the time of testing.

All subjects performed maximal voluntary isometric contractions (MVC) and contractions intending to maximize the rate of moment development (RTD). All contractions were performed in the same hip-knee-ankle joint angle configuration (110-180-90°). Straight hip and knee joint angles were defined as 180°. The shank perpendicular to the foot was defined as 90°. The foot of the participants was placed on the dynamometer’s (HUMAC NORM Model 770; CSMi, Stoughton, MA, USA) footplate adapter. Care was given to align the axis of rotation of the dynamometer to the axis of rotation of the ankle joint. We defined the axis of rotation of the ankle joint to be parallel to the axis of the dynamometer passing through the midpoint of the line connecting both malleoli. In the literature [4,5,15], the firm positioning of the participant’s foot on the dynamometer’s adapter is accompanied by the use of inextensible straps to prevent any ankle joint rotation. In addition to that method, to the best of our knowledge, no study has measured the effect of foot straps on joint mobility during plantar flexion MVC. Therefore, we refrained from securing the foot on the dynamometer plate in order to assess the maximal ankle joint rotation affected only by the positioning of the subject. Consequently, we expected an overestimation of the ankle joint rotation using this method.

Prior to marker placement, participants performed a warm-up phase of approximately 5 minutes where they executed multiple submaximal as well as 2 maximal isometric plantar flexion efforts prior to testing for preconditioning purposes [16]. Following the warm up, the participants were instructed to perform three ramp (3–4 s) maximal isometric voluntary plantar flexion contractions and sustain them for ~4 s. Between trials, the thigh’s non-elastic strap was loosened, and participants were given at least one minute of rest to prevent muscle fatigue or numbness [1,17]. The same investigator tightened the participant’s thigh prior to any MVC using the same procedure. To assess the RTD, the participants were subjected to two additional contractions with a 20-s rest [18] between them. The participants were instructed to develop MVCs as fast as possible. Visual feedback and encouragement [19,20] were given during all the MVC and RTD efforts. The RTD was calculated as the slope between two consecutive time units until the maximal achievable moment was achieved. This method follows the assumption that the tangential RTD could probably display the influence of all contributory parameters before or between that instant [21].

**Fig 1:**
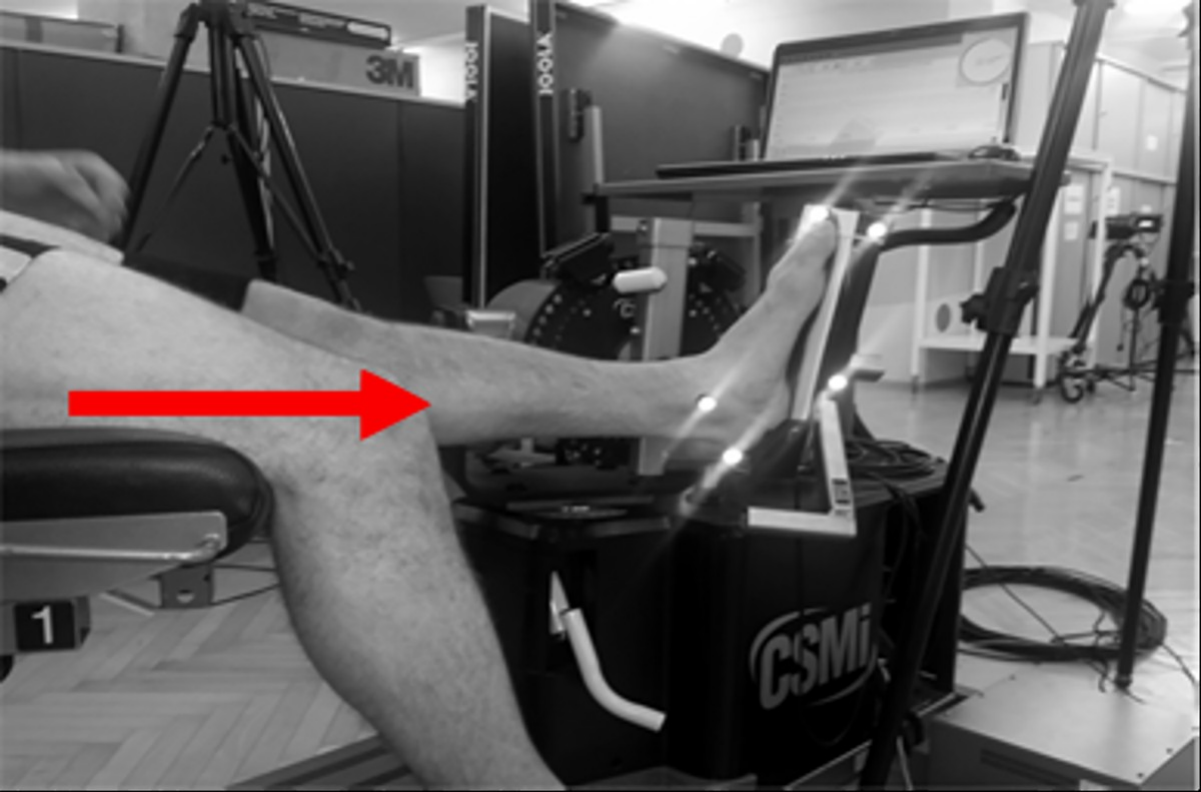
Exemplarily setup for the plantar flexion contractions. The arrow demonstrates the positioning direction of the subject.

The neutral (“0 cm”) position of the subjects was first identified using the same method reported in the literature. The criterion for the neutral position was to establish firm placement of the subject’s foot on the dynamometers plate with both the dynamometer and ankle joint axes aligned. For the subsequent trials, we moved the subject’s chair (Fig 1) 3, 6, and 9 cm toward (anterior) the dynamometer plate. All the MVCs and RTDs contractions (Table 1) at the 4 different dynamometer positions were performed in randomized order. At the most anterior position (“9 cm”), several participants exhibited pain or felt uncomfortable. In these cases, we used the next possible increment (7 or 8 cm); however, for simplicity, in the present manuscript, we referred to this increment as the “8 cm” position.

**Table 1:** Measuring positions and horizontal distances of the dynamometer chair according to the “neutral” position.

Position	Distance to dynamometer axis [cm]		
0	0.00 ± 0.00		
3	3.00 ± 0.00		
6	6.00 ± 0.00		
9	8.25 ± 0.85		

(mean ± SD, n = 14)

Kinematic data were recorded using the Vicon-MX-Motion-Capturing-System (Oxford, UK) with eight cameras operating at 200 Hz. Reflective markers were placed and captured on the following anatomical landmarks: The C7, trochanter major, the most prominent points of the lateral and medial femoral condyles, lateral and medial malleolus, the most prominent point of the tuber calcanei and on the forefoot on the pressure insole between the second and third metatarsals. Markers were also placed on the axis of the dynamometer, and two markers were placed on the footplate of the dynamometer (Fig 1). The midpoint of the lines connecting both the malleoli and femoral condyle were defined as the ankle and knee joint centers. The marker positions were low-pass filtered using a fourth-order, zero phase-lag Butterworth filter with a cutoff frequency of 12 Hz [22].

The joint moment was measured using the HUMAC isokinetic dynamometer, where the analog signal was captured using the Vicon Nexus A/D card (16 bit) at 2000 Hz. Gravitational forces acting on the foot-dynamometer arm system were compensated for all subjects prior to the voluntary contractions. The corrected moment was calculated through inverse dynamics by a method previously [4,5] reported (Eq 1). Briefly, to calculate the lever arm of the reaction force to the ankle joint during the plantar flexion contraction, assuming the force to be perpendicular to the dynamometer footplate, we determined the point of force application under the foot using a flexible pressure distribution insole (Pedar-X; Novel GmbH, Germany) operating at 100 Hz as follows:

$$M_{corr}=Fd_{A}=M_{meas}\frac{d_{A}}{d_{B}},$$ (1)

where M_corr_ is the corrected joint moment, F is the perpendicular force exerted on the dynamometer footplate at the point of force application, d_A_ is the lever arm of the force (F) to the ankle joint defined as the midpoint of both malleoli, d_B_ is the lever arm of the force (F) to the dynamometer axis, and M_meas_ is the moment measured by the dynamometer device.

The synchronization of all systems was established by a custom-made trigger device (TTL, 0–5 V) that was connected to both the Pedar-X and Vicon Nexus systems. By triggering twice, the Pedar-X system started and then stopped. The last trigger signal was used to synchronize all the captured data. All the acquired data (kinematic, pressure) were interpolated using cubic splines to achieve a common (2000 Hz) frequency. The joint moment and pressure data were low-pass filtered with a fourth-order, zero phase-lag Butterworth filter using a cutoff frequency of 10 and 6 Hz, respectively.

The examined parameters of the joint moment, joint angle and footplate rotation were identified and analyzed at 100% of the MVC and that of the foot pressure was analyzed at 0% of the MVC. All the MVCs and maxRTDs were used for statistical analysis assuming normal distributions due to the high number of observations per group [23]. To identify a possible effect of the position on the examined parameters, we conducted one-way ANOVA with repeated measurements. In the case of a significant effect, a post hoc test with Bonferroni correction was performed to identify the differences among the four positions (0, 3, 6 and 8 cm). Additionally, we performed paired t-tests between the corrected and measured moments and Pearson’s correlation between the maximal exerted moment and four different positions. The level of statistical significance was set at p<0.05.

## Results

**Fig 2:**
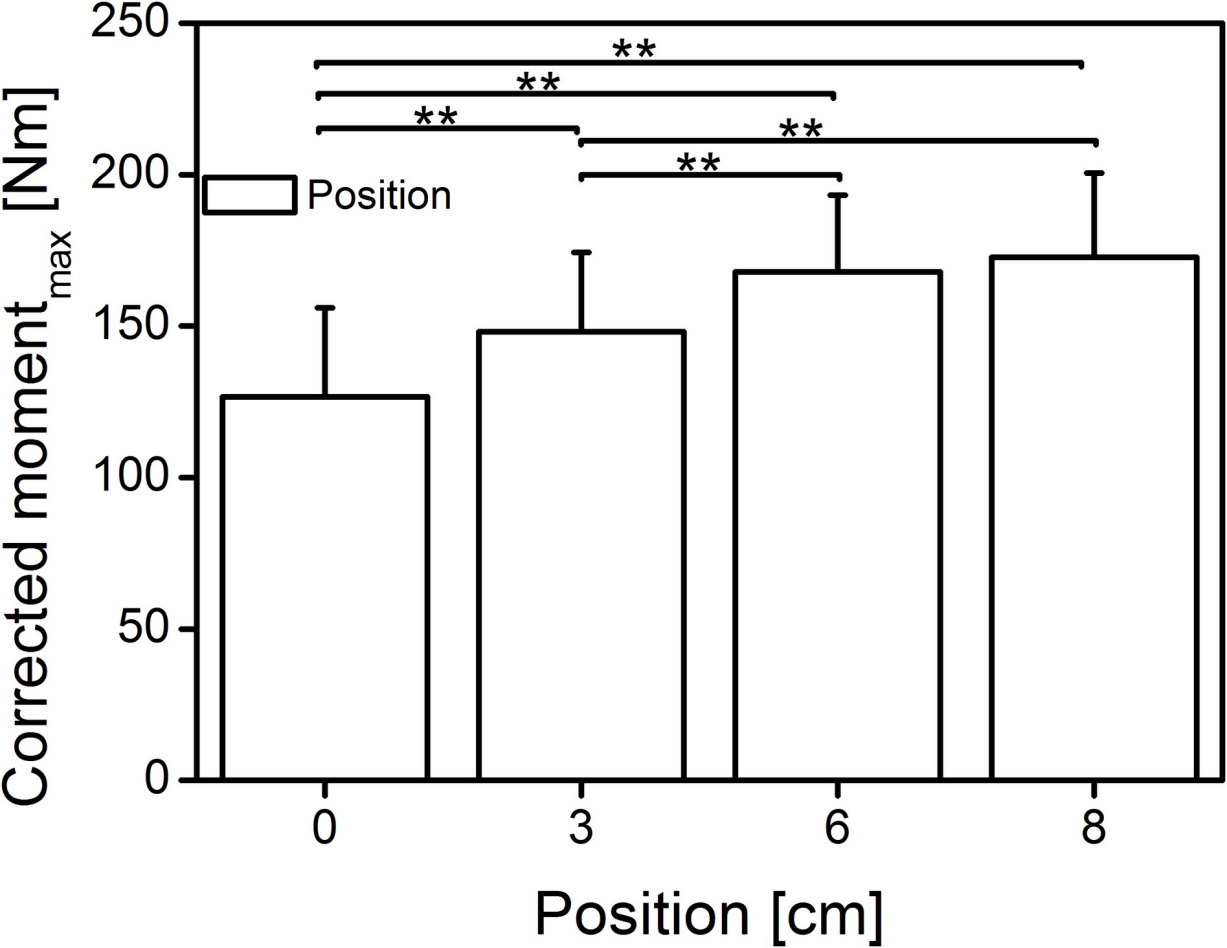
Mean (±SD) of the corrected maximal exerted joint moment at the different positions. The bars indicate significant differences among the positions (0 cm = neutral, 3-6-8 cm; n = 42); **significant difference (p < 0.01).

A significant main positioning effect (Wilk’s lambda = 0.128, F (3, 39) = 88.884, p < 0.001, η^2^ = 0.872) was found (Fig 2) for the corrected joint moment (126.7 ± 29.4, 148.1 ± 26.3 167.9 ± 25.3 and 172.7 ± 27.8 Nm at 0, 3, 6 and 8 cm, respectively). The positions 6 and 8 cm showed significant differences (p<0.001) compared with positions 0 and 3 cm but not (p>0.05) between them.

**Fig 3:**
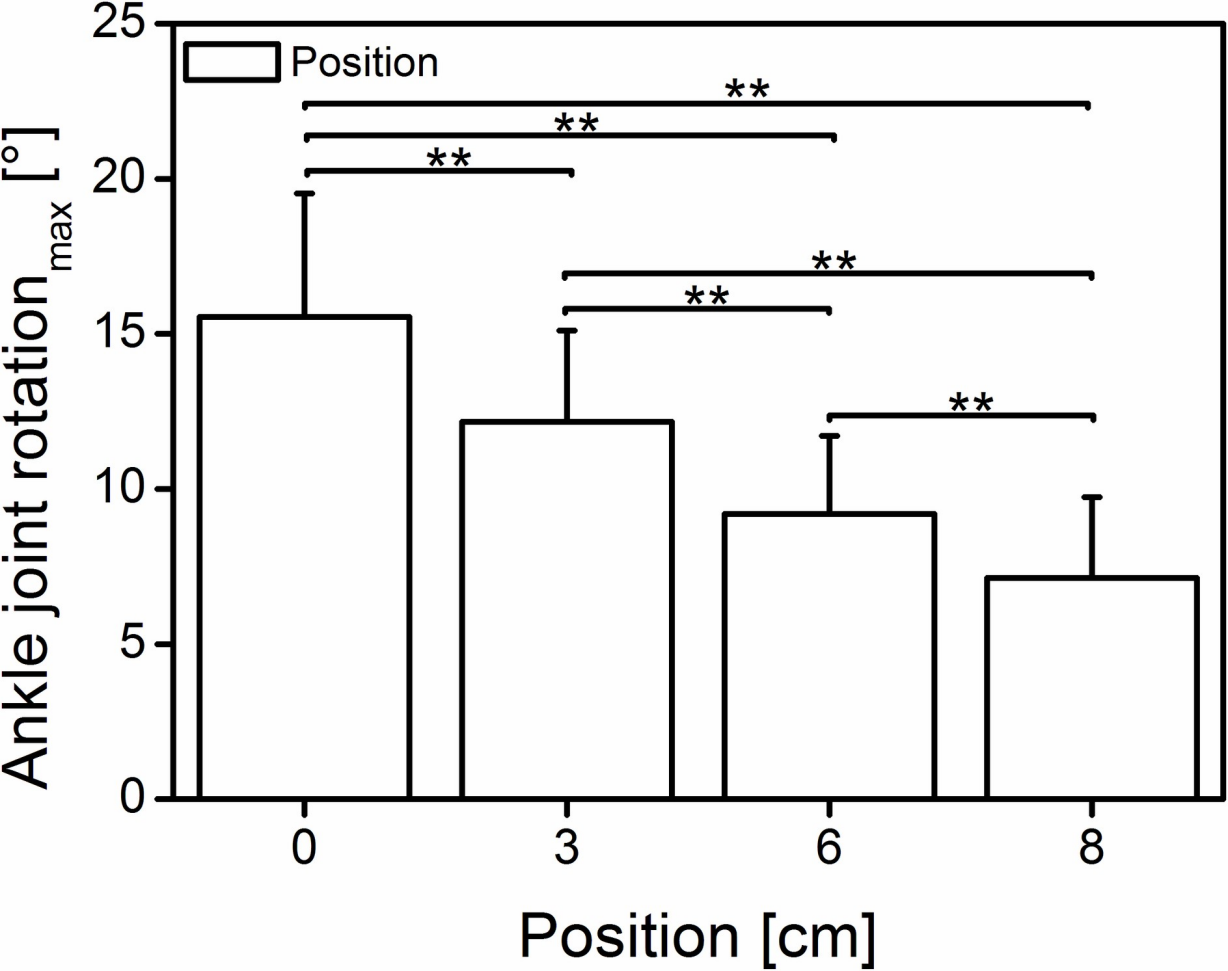
Mean (±SD) of the ankle joint rotation at different positions. Bars indicating significant differences among the positions (0 cm = neutral, 3-6-8 cm; n = 42); **significant difference (p < 0.01).

A significant main positioning effect (Wilk’s lambda = 0.065, F (3, 39) = 185.771, p < 0.001, η^2^ = 0.935) was also found (Fig 3) for the ankle joint rotation (15.5 ± 4.0, 12.2 ± 2.9, 9.2 ± 2.5 and 7.1 ± 2.6° at 0, 3, 6 and 8 cm, respectively). The increased anterior positioning of the subject significantly (p<0.001) reduced the ankle joint rotation ranging from 15.5 to 7.1° (at 0 and 8 cm, respectively).

**Fig 4:**
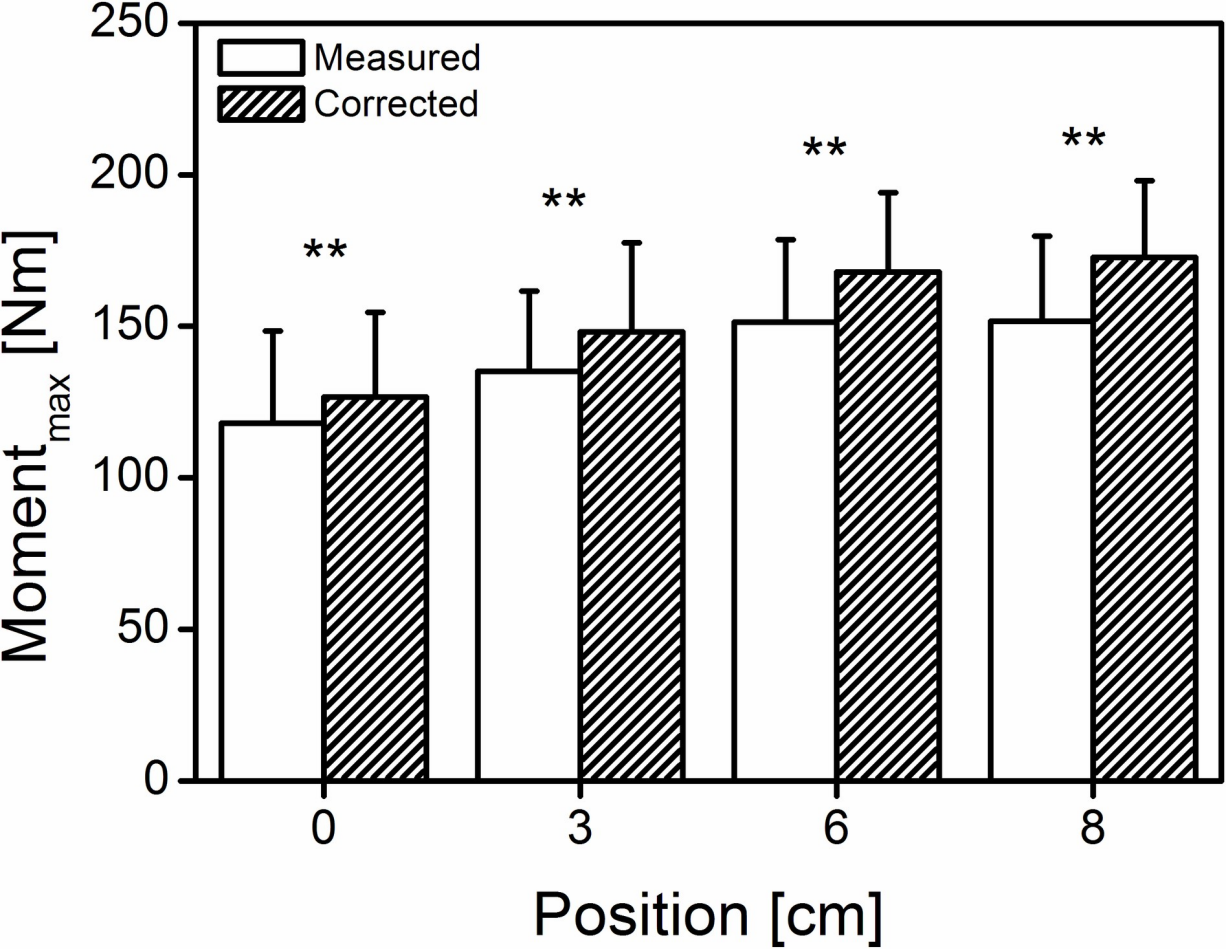
Mean (±SD) of the measured and corrected maximal exerted joint moment. Open box: measured joint moment, shaded box: corrected joint moment at the different subject positions (0 cm = neutral, 3-6-8 cm; n = 42). **significant differences (p < 0.01) between the measured and corrected maximal joint moment.

The T-test for 2 dependent samples revealed a significantly (p<0.01) higher corrected joint moment compared with the measured one (Fig 4) at all four positions (measured moment: 118.0 ± 30.4, 135.2 ± 26.5, 151.3 ± 27.2, and 151.6 ± 28.1 Nm at 0, 3, 6 and 8 cm, respectively).

**Fig 5:**
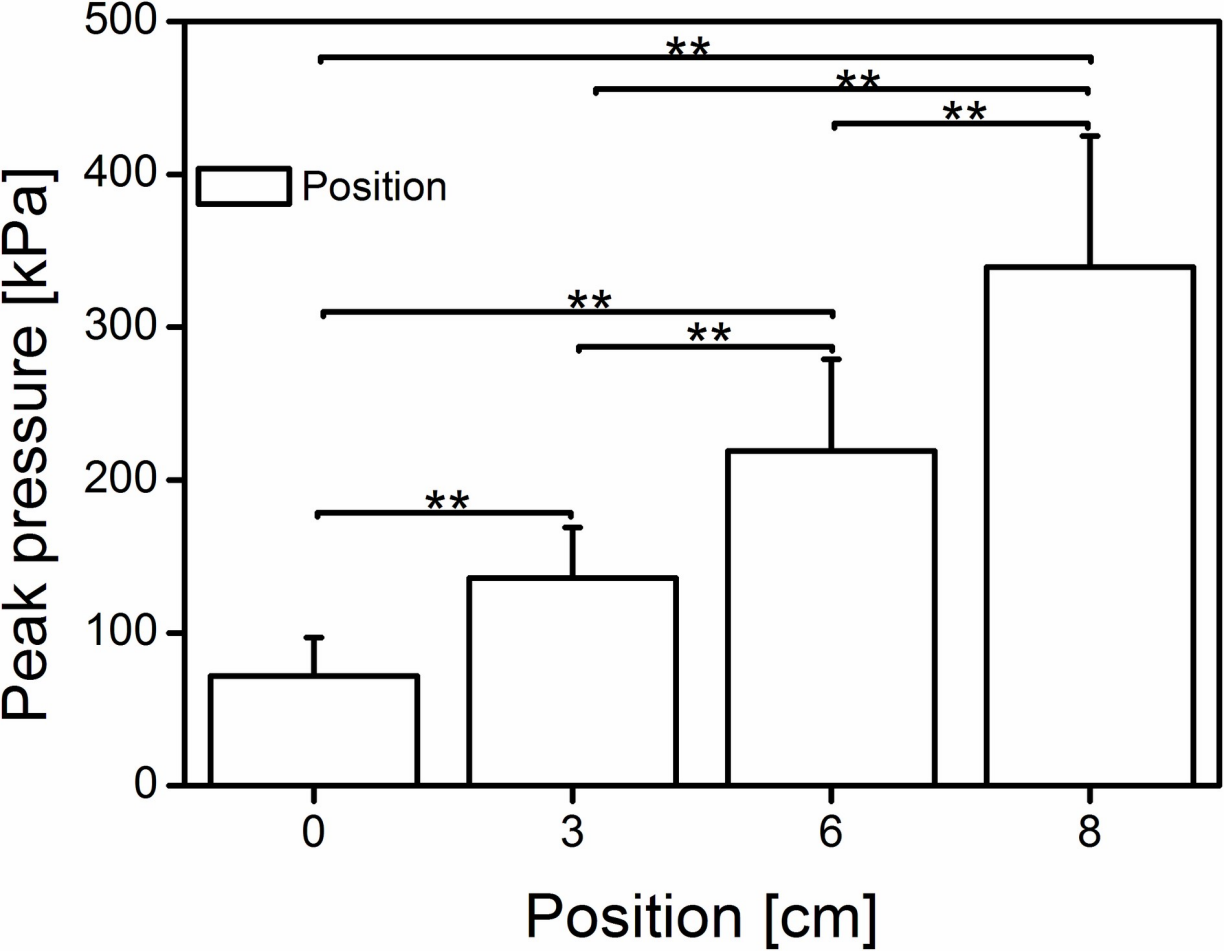
Mean (±SD) of the peak pressure of the subject’s foot on the dynamometer plate at rest. The bars indicating significant differences among the positions (0 cm = neutral, 3-6-8 cm; n = 42); **significant difference (p < 0.01).

As expected, the anterior positioning of the subject increased the peak pressure over 4.7-fold (Fig 5) at position “8 cm” compared with that at position “0 cm” (71.7 ± 25.2, 136.0 ± 32.8, 219.0 ± 60.1, and 339.5 ± 85.6 kPa at 0,3,6 and 8 cm respectively). Statistical analysis revealed a positioning effect (Wilk’s lambda = 0.062, F (3, 39) = 196.005, p < 0.001, η^2^ = 0.938) on the developed foot pressure on the dynamometer plate at rest. The post hoc test showed significant differences (p<0.001) among all positions (Fig 5).

**Fig 6:**
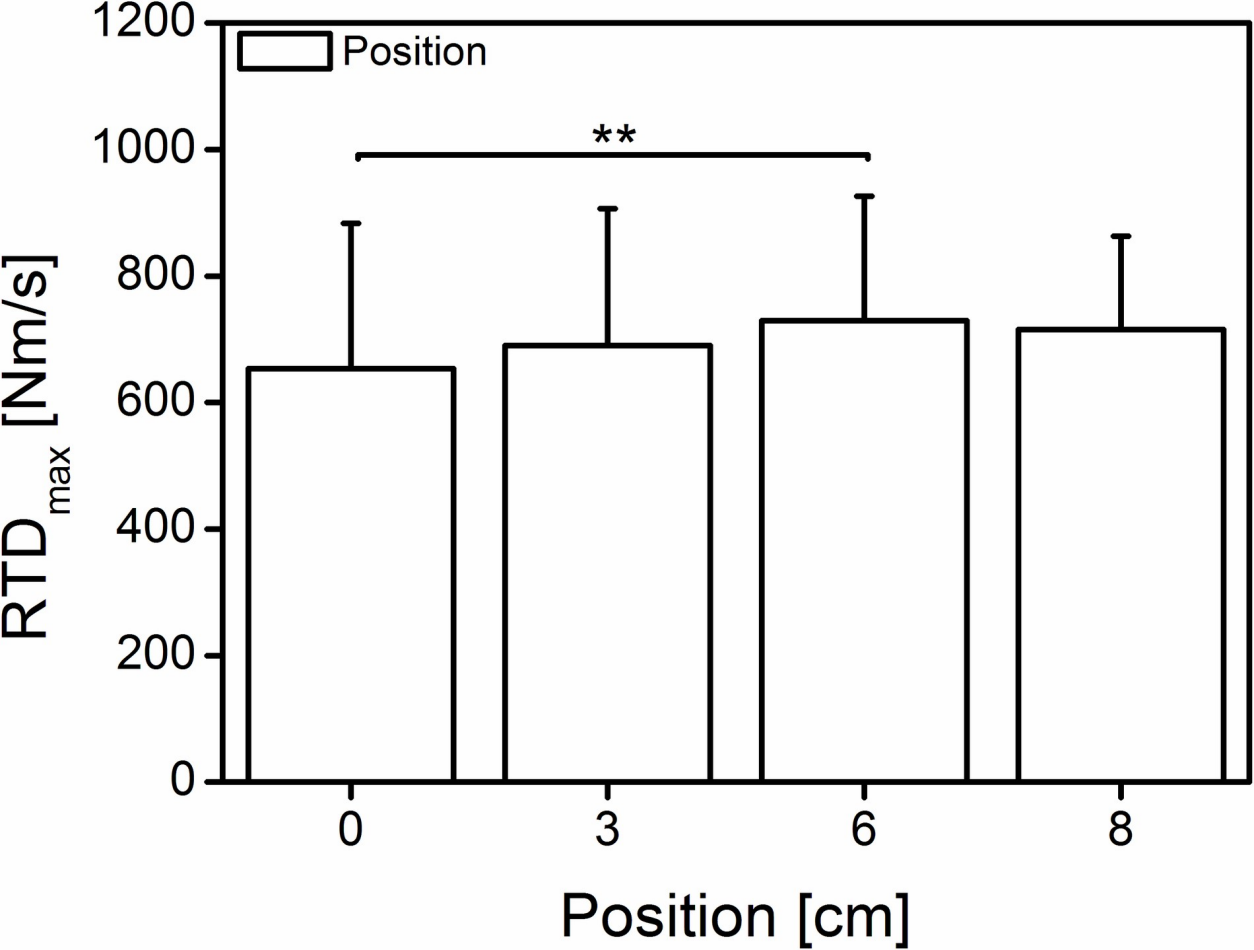
Mean (±SD) rate of torque development at four different positions. The bars indicating significant differences among the positions (0 cm = neutral, 3-6-8 cm; n = 28); **significant difference (p = 0.017).

A significant main positioning effect (Wilk’s lambda = 0.709, F (3, 25) = 3.419, p = 0.033, η^2^ = 0.291) was found (653.2 ± 230.4, 690.4 ± 215.8, 729.7 ± 196.6, and 715.6 ± 147.4 Nm/s at 0, 3, 6 and 8 cm, respectively) in the rate of torque development (Fig 6). The post hoc test showed significant differences (p = 0.017) only between position “0 cm” and “6 cm” (Fig 6).

The hip and knee joint angle were rotated during the MVC efforts (Table 1) with respect to the reference. Statistical analysis showed a significant position effect on the hip joint angle (Wilk’s lambda = 0.268, F (3, 39) = 34.418, p < 0.01, η^2^ = 0.732) and knee joint angle (Wilk’s lambda = 0.666, F (3, 39) = 6.527, p < 0.01, η^2^ = 0.334). Post-hoc analysis revealed significant (p<0.01) knee joint extension and hip joint flexion at 3–6 and 8 cm (Table 1) compared with the “0 cm” position.

## Discussion

The main finding of the present study is the significant increase in the maximal plantar flexion moment with increased anterior positioning of the subject toward the dynamometer footplate. The effect in the most anterior position (>32%) was higher than all the corrections (moment arm, coactivation and joint rotation) combined (6–10%), as presented in the literature [4]. In this study, we attempted to position the subjects using the same method as previously described by other researchers [4–6] who used comparable isokinetic devices. Consequently, the corrected plantar flexion max. moment at the first position (“0 cm”) is in agreement with previous reported values [2,6,7,24] (range: 115–161 Nm) examined in a healthy male population.

### Maximal joint moment

**Fig 7:**
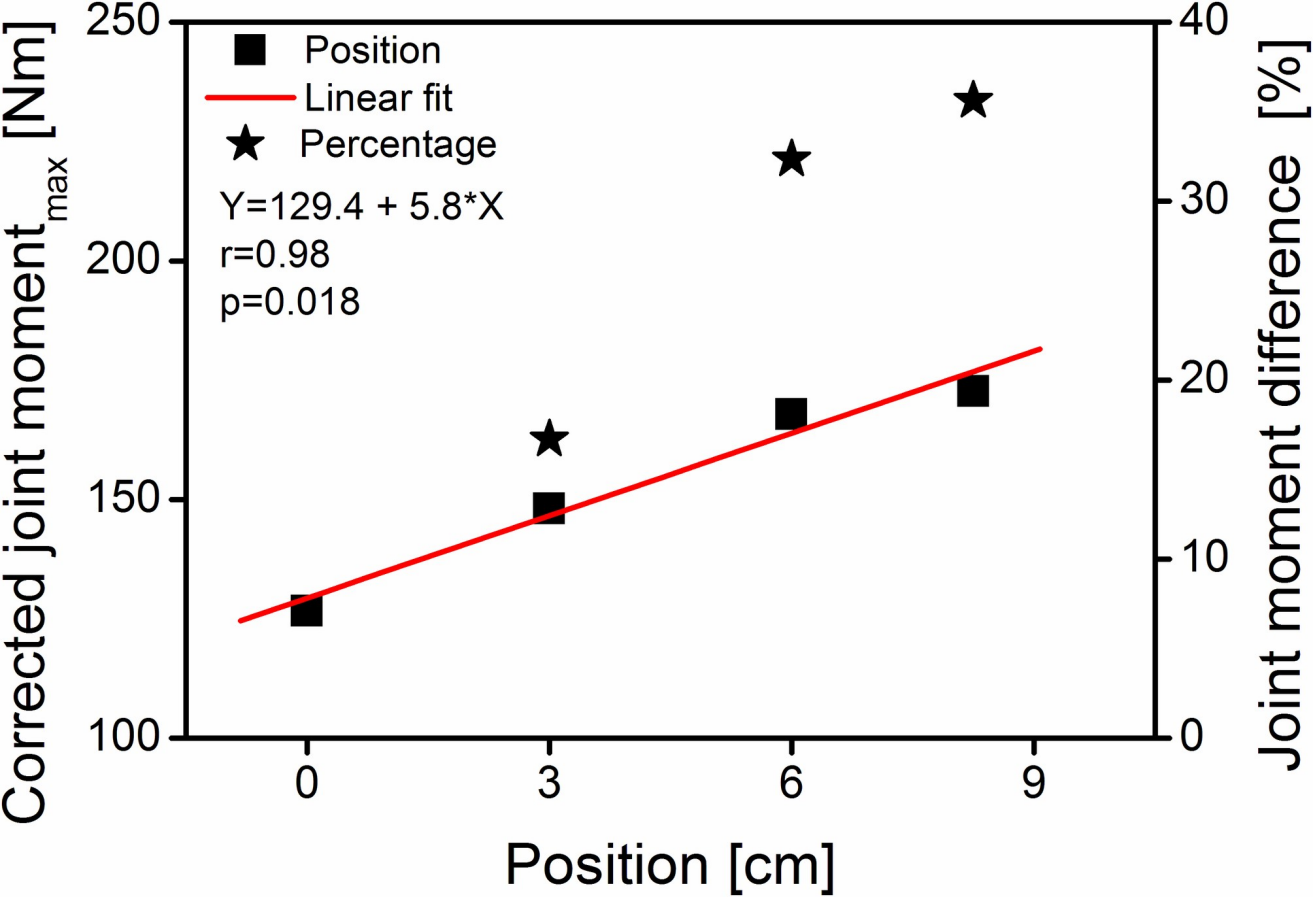
Relationship between the position and the exerted joint moment and percentage difference from position “0 cm”. Mean of the corrected joint moment at four different positions (filled squares). The standard deviation was removed for clarity. The joint moment percentage difference of the 3-, 6, and 8-cm positions from the neutral (“0 cm”) position (filled stars). The maximal corrected joint moment for each of the four positions was linear fitted (red line). There was a significant relationship between the maximal corrected moment (r = 0.98, p = 0.018) and the subject’s position.

The increased moment values were evident in the measured as well as in the corrected joint moment (Fig 4), indicating that measurements dependent on subject positioning are susceptible to error and are crucial in acquiring valid data. Numerous studies [25–27] were planned with an intervention (training or detraining) of the lower extremity muscle tendon unit and examined its effect in a pre-post study design. The present study demonstrated that a potential measurement error exists; thus, it is extremely important, in a pre-post study design, to keep all measurements the same distance of the subject’s chair to the dynamometer footplate. Based on the presented results, a change in position that lies between 0.5 and 2 cm, can induce an error in the maximal exerted moment in the range of 2–9% (Fig 7). This difference is substantial when force increments >2% are expected through an intervention. Furthermore, in our study, the gain in the maximal exerted moment was >32% in the last two positions. The aforementioned joint moment increase denotes that, with commercial dynamometers, a highly probable underestimation of the maximal force generation capacity of the triceps surae exists. Additionally, although at the last position (“8 cm”), several subjects sensed an uncomfortable feeling, the maximal exerted moment was not significantly higher compared to the “6 cm” position, indicating that no further positioning adjustment is needed to achieve the maximum joint moment.

It was previously reported [4] that the measured moment overestimated the resultant moment, a finding that was attributed to the differences between the moment arm of the ankle joint and dynamometer axis to the point of force application. Although we confirmed the findings of Arampatzis et al. (2005), in the present study, the measured moment was underestimated (range: 7–14% from “0 cm” to “8 cm”, respectively) compared with the corrected joint moment (Fig 4). The reason for this discrepancy could be attributed to the positioning of the subject’s foot on the dynamometer footplate. Underestimation of the corrected moment indicates inferior placement of the ankle joint center with respect to the dynamometer axis, resulting in a reduced joint moment arm compared with the moment arm of the dynamometer and vice versa (sitting position, ankle joint at 90°). Nevertheless, the findings of both studies denote the necessity to monitor the kinematics of the ankle joint with respect to the axis of the dynamometer during plantar flexion efforts.

### Foot pressure

To standardize the positioning of the subject in maximal isometric plantar flexion contractions, the use of pressure insoles could reveal fair estimates of the proper position. In the present study, the peak pressure at rest (Fig 5) increased significantly from 219 to 340 kPa (at 6 and 8 cm), although the max. joint moment did not show any significant difference. This finding would indicate that a minimum foot pressure of ~220 kPa is necessary to develop the maximum plantar flexion moment in similar isokinetic dynamometers. Still unanswered is whether that amount of foot pressure would also be necessary for more rigid systems. Further studies are needed to examine the influence of higher foot pressure on the exerted joint moment in rigid dynamometers.

### Ankle joint kinematics

**Fig 8:**
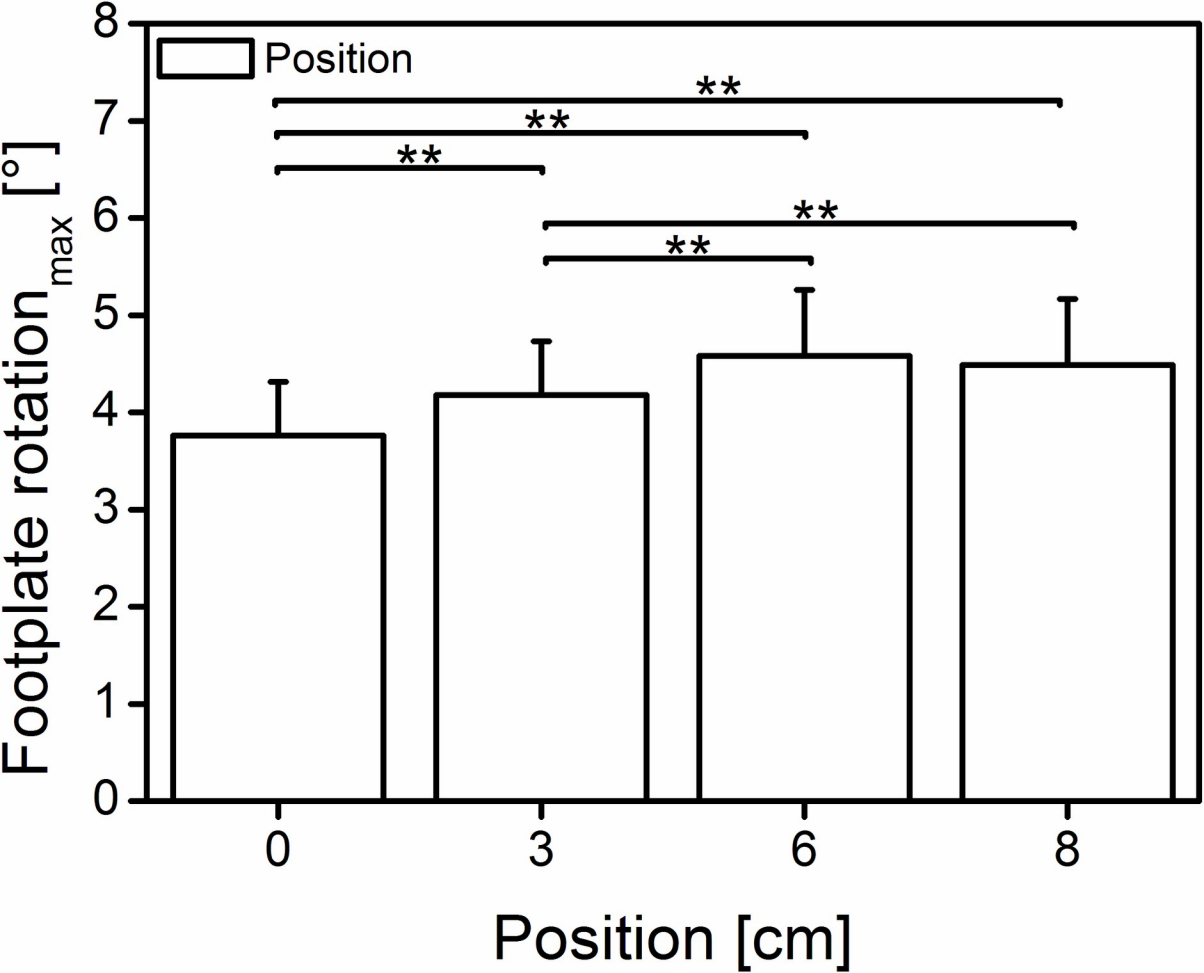
Dynamometer foot plate rotation. Mean (±SD) of the maximal dynamometer plate rotation (3.8 ± 0.5, 4.2 ± 0.6, 4.6 ± 0.7, 4.5 ± 0.7°) at the maximum exerted joint moment on the four different subject positions. The bars indicate significant differences among the positions (0 cm = neutral, 3-6-8 cm; n = 42); **significant difference (p < 0.001).

The ankle joint rotation in the “0 cm” position is in line with previous [28] (17.8°) findings but differs in magnitude from that in other research groups [2,29] (3.2° and 7.4°, respectively), indicating that the magnitude of ankle joint rotation could be dynamometer device dependent. With increased anterior positioning of the subject, the magnitude of joint rotation was reduced by ~54% (from 15.5 to 7.1°) at the last (“8 cm”) position. Further reduction of joint rotation from position “6 cm” to “8 cm” (9.2 and 7.1° respectively) could be partly attributed to further deformation of the seat foam pad because the dynamometer’s foot-plate rotation (Fig 8) did not significantly differ (p>0.05) between both last positions (4.6 and 4.5° respectively). Although the most anterior positioning was sensed as uncomfortable due to the increased pressure, the joint rotation could not be completely avoided, confirming previous findings [4,5,24] that reported an inevitable joint angular rotation during plantar flexion efforts.

Moreover, because the force potential is fascicle length dependent [30], a small joint rotation could further reduce the length change of the fascicles (less shortening), allowing them to operate at their theoretical optimum or desired length. Although we observed similar joint moments (Fig 2), but significantly different joint rotations (Fig 3) at the last two positions, it is unclear whether the fascicle length undergoes any significant changes. Therefore, future studies should examine the fascicle kinematics under similar protocol conditions to identify the possible origin of the increased moment.

### Possible effects on the MTU neuromechanical properties

The force potential of a muscle depends also on neural activation [31]. Particularly, a previous study [6] showed a higher maximal EMG activity toward elongated fascicles. This finding could partially explain the increased maximal moment of the present study because the reduced ankle joint rotation in the last (“6 cm” and “8 cm”) positions could probably reduce the fascicle length shortening of the triceps surae muscles.

Owing to tendon mechanics, an increased joint moment could possibly affect the: a) tendon stress due to the higher forces acting on an approximately similar tendon area, the b) tendon elongation and the c) Young modulus. It is unclear whether the stiffness and hysteresis could also be affected.

### Rate of torque development

**Fig 9:**
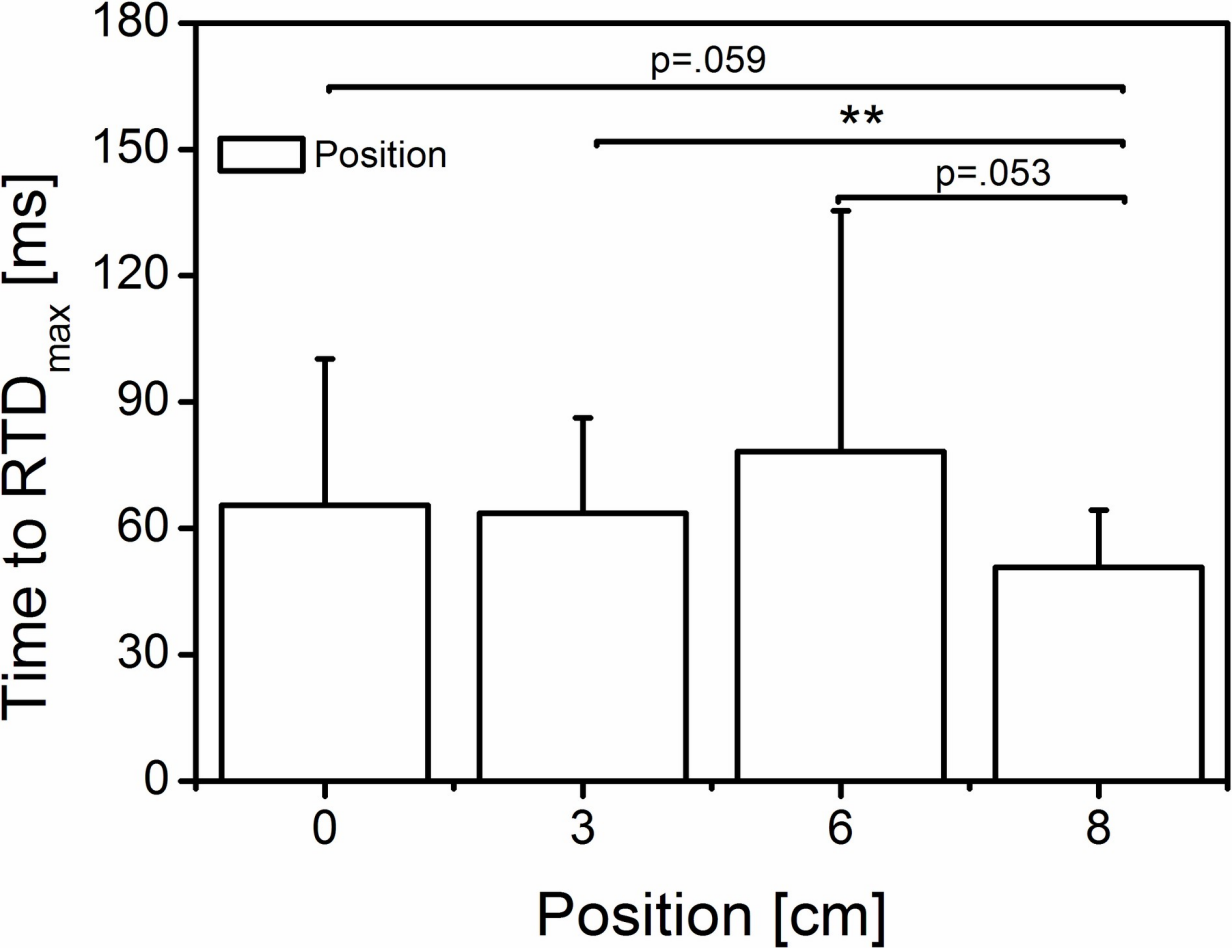
Time to reach the maximal rate of torque development. Mean (±SD) (65.5 ± 34.8, 63.6 ± 22.6, 78.2 ± 57.2, 50.7 ± 13.6 ms for the 0, 3, 6 and 8 cm position, respectively). Statistical analysis showed a significant main position effect (Wilk’s lambda = 0.563, F (3, 24) = 6.202, p = 0.003, η^2^ = 0.437) on time to reach maxRTD. The bars indicate a significant difference and tendency among the positions (0 cm = neutral, 3-6-8 cm; n = 27); **significant difference (p < 0.01).

In the present study, we hypothesized that a higher foot pressure on the measuring device would increase the rate of moment development. It was suggested [9] that dynamometer compliance will allow uncontrolled changes in the joint rotation and velocity [32] that would further decrease the force potential during muscular contraction due to the force-velocity relationship. The current findings (Fig 6) could not support that assumption because, only at the position “6 cm”, did we find a significantly greater RTD, although the maximal exerted moment at “6 cm” and “8 cm” did not significantly differ. According to the previous assumption, due to the joint rotation at position “0 cm” (~15.5°), the RTD should be less than that at position “8 cm” (~7°). It appears that, although the maxRTD was greater at position “6 cm”, there is a tendency for shorter times to reach maxRTD at position “8 cm” (Fig 9). This finding would be in line with the main hypotheses but would not explain the lack of differences between position “6 cm” and “3 cm” and “0 cm”, respectively” (Fig 9). Additional estimations based on the Moment-Time-Integral over the first 50 ms (threshold 7 Nm) of the MVC effort showed no significant positional effect (0.95 ± 0.17, 0.94 ± 0.20, 0.99 ± 0.18, and 0.95 ± 0.16 Nm*s for the 0, 3, 6 and 8 cm position, respectively). From the present findings it remains unclear whether the dynamometer-subject compliance affects the rate of torque development during MVC efforts.

It can be argued that the methodology used to calculate the maxRTD could be susceptible to greater variations [33] and, thus, could have reduced reliability. Because maxRTD can be assessed as the absolute [34–37] or near-absolute [38] rise in the moment per unit of time, we are confident that, using the present method, we could provide valid estimates of the muscle’s mechanical characteristics.

## Limitations

The present findings can be applied to isokinetic dynamometer devices that exhibit similar elasticity. In the present study, we assumed that the force vector excreted under the foot sole is perpendicular to the footplate. In a previous study [4], a difference due to shear forces (2.4°) of 3.8% on the ankle joint was calculated. Nonetheless, it remains unclear how increased are the shear forces with greater foot pressure and at more plantar flexed positions.

It can be argued that bare-foot plantar flexion contraction may increase the uncomforted feeling and, hence, limit the maximal exerted moment, particularly in the last position. We implemented a method used in the past [6,39,40] requiring a bare-foot condition to measure the PoF application and, hence, calculate the moment arm. Nonetheless, we received no complaints in this regard from the participants, and additionally a possible higher moment output on the last position would not change the main findings of the present study.

It can also be argued that the absent of foot strapping can facilitate the joint rotation and reduce the force generation capacity of the triceps surae muscles. In order to assess the influence of foot strapping (SE) on the two parameters we conducted a separate pilot study (S2 File) at the four different positions. The main finding was that the SE plantarflexion moment did not differ compared to the non-foot strapping condition (NSE) at all positions. We found also significant lower (p<0.05) SE moment at 0cm compared with the NSE at the 6cm position. The joint rotation with SE was significant lower (p<0.05) than the NSE condition only at the 0cm position. Those results indicate that the efficacy of the foot strapping in joint rotation reduction is present only in position with low foot pressure on the dynamometer adapter. Therefore, we are confident that the absence of foot strapping in this study would not consist of a limiting factor for the force generation capacity of the triceps surae muscle group.

The knee joint rotation can alter the length of both biarticular gastrocnemii muscles and, hence, their potential to generate force due to the force-length relationship. In our study, the maximal knee joint angle rotation was 2.3° (Table 2), which would represent a fascicle length change of 0.66 mm, and, therefore, its effect on the generated moment can be neglected [41]. The approximately 6° hip joint flexion (Table 2) can be attributed to the compliance of the dynamometers back rest cushioning pad. With increased forward positioning and during the MVC, the trochanter marker was probably moved toward the metal frame of the back rest; consequently, the hip joint angle could not be kept constant. Nonetheless, a hip joint flexion could facilitate a knee joint extension due to the biarticular hamstring muscles. However, in the present study, the knee extension was marginal (2.3°) and could not significantly alter the main findings of this study.

**Table 2:** Hip and knee joint angle rotation at MVC_max_.

	Knee jointmax [°]	Hip jointmax [°]
0 cm	1.0 ± 4.2	-0.1 ± 5.1
3 cm	-0.7 ± 4.7^a^	-3.8 ± 6.0^a^
6 cm	-2.3 ± 4.9^b^	-5.5 ± 5.4^b^
8 cm	-1.9 ± 3.2^c^	-5.7 ± 4.5^c^

Negative values indicating knee extension or hip flexion.

a, b, c, significant difference (p<0.01) from position “0 cm” (mean ± SD, n = 42).

The implemented rest duration of 1 minute between MVC contractions could be a limiting factor for the muscle’s force generation capacity. Nonetheless, in the present study we fully randomized the sitting positions and as a consequence a moderate negative effect of that systematic error could be expected.

It was also previously reported [4] that the contribution of the antagonist m. tibialis anterior to the resulting joint moment was, on average (across different knee and ankle joint configurations), approximately 4.3%. Nonetheless, the attribution of antagonist coactivation (~4.3%) to the corrected joint moment would also not alter the main finding of the present study.

## Conclusion and perspectives

A higher foot pressure may significantly increase the exerted moment and reduce ankle joint rotation during isometric MVC efforts. A minimum pressure of ~220 kPa on the subject’s foot should be developed to achieve the “true” maximum plantar flexion moment. The increased maximal exerted moment could have important implications in longitudinal interventional pre-post study designs when examining various mechanical and morphological properties of the lower leg muscle tendon unit. From the present study, it remains unclear whether the muscle fascicle force-length relationship or muscle activation is responsible for the increased moment. Further studies are needed to identify the mechanisms responsible for the increased moment, focusing on the electromechanical and morphological properties of the human muscle tendon unit.

The implemented flexible pressure distribution insole may not be available for clinical or rehabilitation use due to the high acquisition cost. Additionally, the soft materials of the pressure insoles cannot be subjected to extensive use; therefore, durability issues may arise that could limit the overall availability. Other alternatives, such as small mobile force plates (k-invent) that can be accordingly modified and adapted for the different dynamometer footplate designs, could solve the problem of durability, providing the point of force application with a high acquisition frequency, but could not provide information on the applied pressure. Future studies are needed to develop and implement such devices for biomechanical and clinical use.

## Supporting information

S1 File S1_File includes all relative raw data. The file contains the raw data of all participants and trials in the four positions in the following order: foot pressure, ankle joint rotation, foot plate rotation, measured max. moment, corrected max. moment and additional max. rate of torque development and time to reach max. rate of torque development.
(XLSX)

S2 File S2_File includes a brief description of the pilot study. The file contains the main hypotheses, method and results of the pilot study. Joint moment and joint rotation are presented in (Figs 1 and 2) and additionally all the data can be found on the Table 1.
(DOCX)
