# Supplementary material for: Anterior subject positioning affects the maximal exerted isometric plantar flexion moment
Source: PLoS One. 2019 Jul 22;14(7):e0219840. doi: 10.1371/journal.pone.0219840 (PMC6645493; doi:10.1371/journal.pone.0219840)
Supplement: S2 File — The file contains the main hypotheses, method and results of the pilot study. Joint moment and joint rotation are presented in (Figs 1 and 2) and additionally all the data can be found on the Table 1. (DOCX) [file pone.0219840.s002.docx]

***PILOT STUDY (brief description)***

***Hypotheses:*** *We tested the hypotheses that foot strapping is more effective, compared to forward positioning of the dynamometers chair (increasing foot pressure), in both joint moment increase and joint rotation decrease during maximal voluntary isometric plantar flexion contractions*

***Method****: Six staff members (2 females: 1.78±0.04m, 56.5±12.0Kg, 22.5±0.7years, and 4 males: 1.81±0.08m, 83.3±17.5Kg, 35.7±9.4years) performed two plantarflexion contractions (knee joint 180°, ankle joint 90°) at 4 different positions (0-3-6 and 8.2±1cm) in two different foot conditions: a) secured (SE) and b) non-secured (NSE). Position and condition were fully randomized. One-minute rest between contraction was given. The average value of the two contractions was taken for analysis. Ankle joint motion (120Hz) was captured synchronously with the dynamometer analog data (1200Hz). Kinematic data were interpolated (cubic spline) to achieve a common frequency. Kinematic and kinetic data were filtered with a 4^th^ order Butterworth zero-lag filter with a cut-off frequency of 12 and 10Hz respectively*

*A nonparametric Wilcoxon's signed-rank test was conducted to compare moment or joint rotation between conditions and positions.*

***Results:*** *No significant moment difference was found between foot conditions (Fig. 1) at the all positions. A significant (p<0.05) lower moment was found in the SE-0cm to the NSE-6cm. We found significant (p<0.05) lower moment of NSE condition at 0cm compared to 6 and 8cm. We also found significant (p<0.05) lower moment of NSE-3cm compared to NSE-6m (Fig. 1).*

*Figure 1: Mean (± SD) of plantarflexion moment at four different positions (0-3-6-8cm) and two different conditions (secured, non-secured).*

*Bars indicating significant differences (p<0.05) between NSE positions.*

**: indicates significant difference (p<0.05) of SE-0cm and NSE-6cm (n=6).*

*The ankle joint rotation was significantly lower (p<0.05) in the SE compared to the NSE condition only at the position 0cm. Foot strapping reduced significant (p<0.05) the ankle joint rotation in positions 6cm and 8cm compared to position 0cm and 3cm (Fig.2). Also the rotation of the NSE foot at position 6cm and 8cm was significant lower than in the SE condition at the position 0cm and 3cm (Fig.2). All values are presented in Table 1.*

*Figure 2: Mean (± SD) of ankle joint rotation at four different positions (0-3-6-8) and two different conditions (secured, non-secured).*

*Bars indicating significant differences (p<0.05) between SE conditions at different positions.*

**: indicates significant difference (p<0.05) between SE and NSE condition at 0cm position.*

*#: indicates significant difference (p<0.05) between SE-0cm and NSE-6-9cm.*

*§: indicates significant difference (p<0.05) between SE-3cm with NSE-6-9cm. (n=6)*

*Table 1: Mean (± SD) plantar flexion moment and ankle joint rotation at four different positions (0-3-6-8cm) and conditions (SE, NSE)*

|  | Moment [Nm] | | Rotation [°] | |
| --- | --- | --- | --- | --- |
| Position | SE | NSE | SE | NSE |
| 0 | 145.3±31.1 | 133.0±41.4 | 10.4±2.4 | 13.6±3.1 |
| 3 | 152.4±26.6 | 147.1±34.9 | 10.0±1.8 | 9.6±2.0 |
| 6 | 155.9±25.9 | 160.4±35.2 | 6.8±3.3 | 5.5±1.9 |
| 8 | 158.5±31.0 | 155.4±34.9 | 5.7±2.2 | 5.9±1.9 |
